# A critical role of affective content in the analgesic effect of virtual reality: a cross-sectional within-subject study

**DOI:** 10.1016/j.lana.2026.101385

**Published:** 2026-02-16

**Authors:** Nandini Raghuraman, Roni Shafir, GianCarlo Colloca, Craig Kier, Barbara Brawn, Amitabh Varshney, Sarah Murthi, Yang Wang, Luana Colloca

**Affiliations:** a Department of Pain and Translational Symptom Science, School of Nursing, University of Maryland, Baltimore, MD, USA; b Placebo Beyond Opinions Center, School of Nursing, University of Maryland, Baltimore, MD, USA; c University of Colorado School of Medicine, Department of Psychiatry, Aurora, CO, USA; d Towson University, Information Technology Program, Towson, MD, USA; e School of Music, University of Maryland College Park, MD, USA; f Department of Computer Science, Institute for Advanced Computer Studies, University of Maryland, College Park, MD, USA; g Department of Computer Science, University of Maryland, College Park, MD, USA; h Division of Trauma and Critical Care, R Adams Cowley Shock Trauma Center, University of Maryland, School of Medicine, Baltimore, MD, USA; i Center to Advance Chronic Pain Research, University of Maryland, Baltimore, MD, USA

**Keywords:** Cognitive engagement, Attention, Emotional regulation, Distraction, Skin conductance, Ambient sound

## Abstract

**Background:**

The dual challenges of the opioid crisis and the global burden of chronic pain underscore the need for safe, non-pharmacological alternatives. Virtual reality (VR) is a promising digital therapeutic for pain, yet its mechanisms remain unclear. This study aimed to disentangle the roles of immersion and emotional engagement in VR-induced analgesia in individuals with temporomandibular disorders (TMD).

**Methods:**

In a counterbalanced within-subject design, 62 adults with TMD (21 males, 41 females; mean age 34.7 years [19–55]; 57 (91.9%) non-Hispanic or Latino, 30 (48.4%) White) were exposed to seven conditions: three immersive VR environments (ocean, opera, pink noise), matched non-immersive (2D) versions, and a 2-back working memory task. Heat pain tolerance was assessed using thermal stimulation. Participants rated pain intensity, pain unpleasantness, mood, anxiety, and enjoyment. Skin conductance response (SCR) indexed autonomic arousal. Multilevel mediation models tested the underlying psychological mechanisms.

**Findings:**

VR Ocean significantly increased heat pain tolerance (Cohen's d = 1.60), reduced pain intensity and unpleasantness, improved mood, and reduced situational anxiety relative to all other conditions (all p < 0.05). It was also rated as the most enjoyable experience (p < 0.01). Mediation analyses indicated mood (ab = −5.15) and enjoyment (ab = −6.12) significantly mediated VR Ocean's effect on pain intensity, whereas anxiety did not. No mediators explained changes in pain tolerance. SCR did not differ between VR and 2D conditions.

**Interpretation:**

VR-based analgesia relies not only on immersion but also on affectively rewarding contents. Digital therapeutics that enhance positive mood and enjoyment may be especially effective for chronic pain management.

**Funding:**

This study was supported by the 10.13039/100000002National Institutes of Health and the University of Maryland.

**Research in context**

**Evidence before this study**

Prior to conducting this study, we performed a systematic search of the literature to identify relevant evidence on virtual reality (VR)–based analgesia and pain modulation. We searched PubMed, Web of Science, and PsycINFO from inception through June 2025. Search terms included combinations of “virtual reality,” “VR,” “pain,” “analgesia,” “immersive,” “2D,” “temporal mandibular disorder,” “TMD,” “mood,” “anxiety,” and “enjoyment.” References of included articles were also screened for additional relevant studies. This search informed the design, selection of experimental conditions, and outcome measures for the present study.

**Added value of this study**

In a counterbalanced within-subject design including immersive and non-immersive versions of three audiovisual environments and a cognitive distraction task, we directly compared the roles of immersion and content in individuals with chronic TMD. We found that a calming, nature based VR environment (VR Ocean) produced the greatest improvements in pain tolerance, pain intensity, unpleasantness, mood, and anxiety. Multilevel mediation analyses showed that positive mood and enjoyment but not anxiety reduction or physiological arousal, mediated VR effects on pain intensity. This is one of the first studies to identify psychological mechanisms underlying content-specific VR analgesia in chronic pain.

**Implications of all the available evidence**

Our findings indicate that VR analgesia is driven not only by immersion but also by emotionally rewarding content. Nature-based, affectively positive VR environments may optimize digital therapeutics for chronic pain by enhancing mood and engagement. These insights can inform VR design principles and support innovative digital, non-pharmacological pain therapeutics during the ongoing need to reduce reliance on pharmacological treatments.

## Introduction

The ongoing opioid crisis has amplified the urgency for effective non-pharmacological alternatives to pain management. Among these, virtual reality (VR) has shown promise in reducing pain across experimental, acute, and chronic settings. Immersive VR, which offers a strong sense of presence in a digital environment, is thought to reduce pain by diverting attention and decreasing the cognitive resources available for pain processing.^1^^,^^2^ Indeed, studies have shown that immersive VR increases pain tolerance compared to non-immersive formats with identical content.^3^, ^4^, ^5^

Yet, immersion alone may not fully account for VR's analgesic effects. The emotional and attentional qualities of the content particularly relaxing, arousing and neutral sounds, may also modulate pain perception. Research in music and sound psychology consistently shows that emotionally pleasant music (e.g., ambient sound) reduces pain intensity and unpleasantness, enhances mood, and lowers anxiety.^6^ However, the integration of such content into immersive VR pain interventions remains limited. Our previous work showed that a nature-based immersive VR environment (“VR Ocean”) enhanced heat pain tolerance and mood in healthy participants, while an opera-based VR environment was less effective, except for participants who reported enjoying opera.^3^ These findings suggest that the affective tone and personal relevance of VR content moderate its analgesic effects. Our prior work also compared immersive natural VR environments (e.g., VR Ocean) to various control conditions (sham VR, non-intervention, neutral content) in healthy participants, showing that externally generated, immersive, and emotionally pleasant content more effectively enhanced pain tolerance than internally self-generated imagery.^5^^,^^7^

We capitalized on sound-based content in our VR study because sound plays a crucial role in shaping emotional, cognitive, and sensory experiences.^8^ We also tested if the effect of VR content on mood is critical to its analgesic effects.^9^ Calming, low-arousal sounds, such as ambient ocean sounds, have been shown to reduce physiological arousal, enhance positive affect, and facilitate pain relief, particularly when paired with nature-based visual environments. In contrast, complex or emotionally intense sounds (e.g., opera) can produce mixed affective responses across individuals.

Building on these results, the present study aimed to examine systematically how the quality of sound-based VR content influences pain-related outcomes and affect in individuals with chronic temporomandibular disorders (TMD), a prevalent and underserved condition affecting up to 32% of the population.^10^ In this observational cross-sectional within-subjects counterbalanced study design, 62 individuals with chronic TMD completed seven experimental conditions: three immersive VR environments (calming ambient sound, arousing opera, neutral pink noise), three corresponding 2D non-immersive controls, and a 2-back working memory task (a distraction manipulation). The auditory stimuli (calming ambient sound, arousing opera, and neutral pink noise) were intentionally paired with distinct visual environments (nature scenes, opera actors, and geometric shapes) to investigate how the VR content influences pain perception and mood. We assessed heat-based pain tolerance (primary outcome), and secondary outcomes such as self-reported pain experience, mood, anxiety, enjoyment, and physiological arousal via skin conductance response (SCR).^11^, ^12^, ^13^, ^14^ We hypothesized that immersive VR with ambient ocean sound would induce the strongest analgesic and mood elevating effects and that the latter effect would be critical to the analgesic effect of immersive VR.

## Methods

### Participants

Sixty-eight adults (41 females) diagnosed with temporomandibular disorder (TMD) participated in this cross-sectional, within-subject study. Participants were recruited using a convenience sampling strategy between March 2019 and May 2020 at the University of Maryland School of Nursing (refer to Supplementary Figure S1). Two participants were lost to follow-up, and four were excluded for missing primary outcome data, leaving 62 adults with chronic TMD (21 males, 41 females; mean age 34.7 years [19–55]; 2 (3.2%) Hispanic, 57 (91.9%) non-Hispanic or Latino, and 3 (4.8%) unknown; 30 (48.4%) White, Table 1) with chronic orofacial pain (≥3 months) meeting established TMD diagnostic criteria.^15^^,^^16^ Participants were diagnosed with TMD according to Axis I Diagnostic Criteria (DC/TMD) following a standardized clinical evaluation at the University of Maryland School of Dentistry. Screening procedures, clinical assessments, and exclusion criteria are detailed in the Supplementary Materials. Ethnicity and race were self-reported, with participants selecting from predefined categories (non-Hispanic or Latino, Hispanic or Latino, Unknown/Prefer not to answer). Race included NIH-defined categories (American Indian or Alaska Native, Asian, Black or African American, Native Hawaiian or Other Pacific Islander, White, and Unknown/Prefer not to answer).

**Table 1:** Sociodemographic, clinical, and psychological characteristics of study participants.

Characteristic	N or mean ± SD	% or 95% CI
**Age (years)**	34.69 ± 10.21	32.10, 37.29
**Sex**		
Male	21	33.9
Female	41	66.1
**Ethnicity**		
Hispanic or Latino	2	3.2
Non-Hispanic or Latino	57	91.9
Unknown	3	4.8
**Race**		
Asian	3	4.8
African American or Black	25	40.3
White	30	48.4
Mixed	4	6.5
**Combined annual household income**		
$0–$19,999	13	21.0
$20,000–$39,999	12	19.4
$40,000–$59,999	9	14.5
$60,000–$79,999	9	14.5
$80,000–$99,999	6	9.7
$100,000–$149,999	6	9.7
$150,000 or higher	7	11.3
**Highest level of education**		
College graduate	30	48.4
Post-graduate	11	17.7
Some college	17	27.4
High school	4	6.5
**Marital status**		
Divorced	3	4.8
Living as married	1	1.6
Married	11	17.7
Never married	43	69.4
Separated	3	4.8
Widowed	1	1.6
**TMD type**^15^^,^^16^		
Myalgia	61	98.4
Myofascial pain with referral	4	6.5
Right arthralgia	50	80.6
Left arthralgia	40	64.5
TMD headache	32	51.6
**Pharmacological treatments**		
Non-steroidal Anti-inflammatory drug	17	27.4
Muscle relaxants	4	6.5
Benzodiazepine	2	3.2
Tricyclic antidepressants	0	0.0
Selective serotonin reuptake inhibitors	10	16.1
Selective serotonin-norepinephrine reuptake inhibitor	3	4.8
Serotonin antagonist and reuptake inhibitors	1	1.6
Norepinephrine–dopamine reuptake inhibitor	1	1.6
**Blood pressure (mm Hg)**		
Systolic	122.55 ± 13.74	119.06, 126.04
Diastolic	77.06 ± 13.11	73.73, 80.39
**Heart rate (bpm)**	75.98 ± 12.59	72.79, 79.18
**Body mass index**	30.30 ± 8.69	28.09, 32.51
**Graded chronic pain scale**^17^		
Pain intensity	49.83 ± 22.40	44.14, 55.52
Pain interference	31.34 ± 28.48	24.10, 38.57
Pain grade (1–2a)^18^^,^^19^: low-impact pain	37	59.6
Pain grade (2b–4)^18^^,^^19^: high-impact pain	25	40.4
**Jaw function limitation scale-20**^20^	2.29 ± 2.18	1.58, 2.69
**Oral behavior checklist**^21^	29.94 ± 12.84	26.67, 33.20
**Pain duration** (months)	124.92 ± 114.52	35.83, 154.01
**Chronic overlapping pain conditions**^22^		
Knee pain	7	11.3
Shoulder pain	5	8.1
Low back pain	20	32.3
Osteoarthritis	9	14.5
Fibromyalgia	1	1.6
Headache	43	69.4
Migraine	10	16.1
Irritable bowel syndrome	4	6.5
**Mood disorders assessments**		
Beck depression inventory score^23^	27.48 ± 23.19	21.59, 33.37
State-Trait anxiety inventory score^24^^,^^25^	41.92 ± 10.87	39.16, 44.68
Depression anxiety stress scale (depression) score^26^	8.06 ± 8.69	5.86, 10.27
Depression anxiety stress scale (anxiety) score^26^	5.65 ± 6.16	4.08, 7.21
**Emotion regulation scale**^27^		
Cognitive reappraisal	30.98 ± 6.19	29.41, 32.56
Expressive suppression	14.50 ± 5.60	13.08, 15.92
**Barcelona music reward questionnaire**^28^		
Emotional evocation	16.37 ± 2.45	15.74, 16.99
Sensory motor	14.41 ± 2.06	13.89, 14.94
Mood regulation	17.00 ± 2.11	16.46, 17.53
Musical seeking	12.06 ± 2.79	11.35, 12.77
Social reward	15.17 ± 3.29	14.34, 16.01
**Game addiction**^29^	59	95.16
Users' experience	39	62.9

### Study procedures

Following informed consent, participants attended a single ∼2-h session at the University of Maryland School of Nursing. Baseline physiological measures (heart rate, blood pressure, height, weight, BMI) were obtained. Skin conductance response (SCR) was recorded during each condition using BrainAmp electrodes on the palm and analyzed with LedaLab software. Pain sensitivity was measured using a thermode on the dominant forearm to identify individualized thresholds (warmth, pain onset, tolerance) and repeated across seven experimental conditions.

**Fig. 1:**
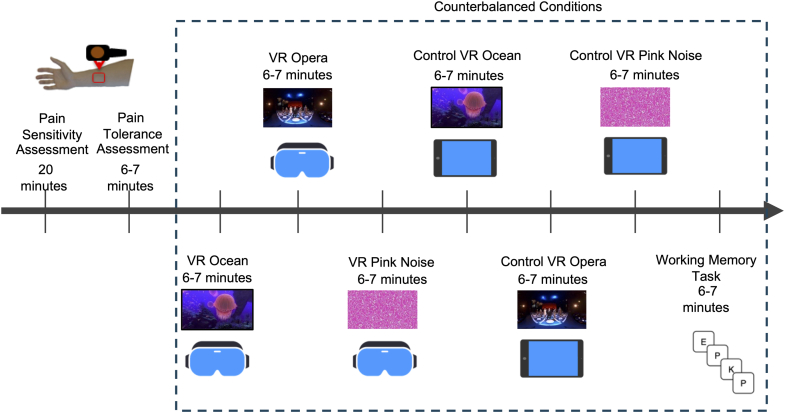
**Study timeline and experimental conditions.** The study followed a within-subject, counterbalanced design to examine pain tolerance across seven conditions. Each participant first completed baseline heat pain assessments on the forearm using a thermode. Participants then underwent seven conditions presented in randomized order: (1) immersive VR Ocean (ocean visuals with ambient sounds), (2) immersive VR Opera (classical opera performance), (3) immersive VR Pink Noise (geometric visuals with pink noise), (4) 2D Ocean, (5) 2D Opera, (6) 2D Pink Noise, and (7) a 2-back working memory task (WMT). Skin conductance responses (SCRs) were recorded continuously during each condition.

Participants completed a counterbalanced within-subject protocol comprising three immersive VR conditions (Ocean, Opera, Pink Noise), three matched non-immersive 2D tablet controls, and a distraction-based 2-back working memory task (Fig. 1). VR content was delivered via HTC Vive Pro and presented immersive visuals with matched auditory components (refer to Supplementary Materials for technical details). No participants reported adverse effects or dropped out due to VR discomfort.

### Experimental conditions and timeline

Participants completed seven counterbalanced conditions in a within-subject design: three immersive VR environments (Ocean, Opera, Pink Noise); their matched non-immersive 2D tablet controls; and a cognitive distraction task (2-back working memory). Each VR environment combined a distinct audiovisual experience with either natural ambient sounds (Ocean), classical opera (La Clemenza di Tito), or repetitive pink noise and abstract visuals. The control conditions matched these environments in content but were presented in 2D using a tablet and headphones, removing immersive features. The 2-back task presented letter sequences on a monitor to assess distraction-related effects. See Supplementary Materials for full stimulus specifications and delivery systems.

The order of the seven experimental conditions was counterbalanced across participants using five MATLAB-generated randomization sequences, ensuring each condition appeared equally often in each position. Participants were randomized to start the experiment under different conditions, and a 6–7 min rest period was provided between sessions to minimize carryover and expectancy effects.

### Primary and secondary outcomes

The primary outcome was heat pain tolerance, computed as the mean temperature (°C) reached across four tolerance trials during each condition. Secondary outcomes included both behavioral ratings of pain intensity, pain unpleasantness, mood, situational anxiety, and enjoyment and physiological assessments such as SCR.

After each condition, participants used a 0–100 visual analog scale (VAS) to rate these dimensions (e.g., 0 = “no pain,” “not unpleasant,” “not anxious,” “extremely bad mood,” “not enjoyable”; 100 = “maximum tolerable pain,” “very unpleasant,” etc.). VAS data were collected via Eprime v2 and a Celeritas Fiber Optic Response System. Individual differences in music reward, emotion regulation, mood, and anxiety were assessed post-session using validated questionnaires (Table 1 and Supplementary Materials). Participants also selected the condition they found most helpful for reducing the unpleasantness of pain and enhancing perceived control.

### Statistical analysis

An independent analyst, blinded to study design and data collection, performed all analyses using SPSS v24 (SPSS Inc., Chicago, IL). Primary and secondary outcomes were analyzed using linear mixed models (LMMs) with participant ID as a random intercept to account for within-subject correlation. Covariates included age, sex, race, and randomization sequence. Exploratory analyses disaggregated by sex/gender and race/ethnicity were conducted for the primary outcome (heat pain tolerance) to assess potential subgroup-specific effects. We implemented two complementary LMM strategies: 1. A 7-condition categorical LMM (primary analysis) and 2. A Factorial LMM excluding the mental task (secondary/mechanistic analysis).

All seven within-subject conditions (Immersive VR: Ocean, Opera, Pink noise; Non-immersive 2D: Ocean, Opera, Pink noise; Mental task) were entered as a single categorical fixed factor. This approach provides a global assessment of condition effects and allows direct pairwise comparisons among any two conditions (including comparisons involving the mental task), while preserving participant-level clustering.

To test mechanistic hypotheses about immersion and auditory content, we fitted a second LMM restricted to the six auditory conditions with Immersion (immersive vs non-immersive), Auditory content (ocean, opera, pink noise), and their interaction as fixed effects. The mental task was excluded from this factorial model because it lacks auditory content; restricting the model in this way yields cleaner tests of immersion × sound interactions.

Effect estimates are presented as estimated marginal mean differences with 95% confidence intervals. Pairwise contrasts and estimated marginal means (EMMs) were computed using the emmeans package and adjusted for multiple comparisons (Bonferroni). LMMs were estimated with maximum likelihood, which accommodates missing data under the missing-at-random assumption.

Missing repeated values were handled in the mixed-effects model. As a sensitivity analysis, we also ran multiple imputation (fully conditional specification with predictive mean matching, 40 imputations) for all outcomes and covariates.

Outlier sensitivity analyses (Tukey's rule) did not materially change results. SCR data (n = 41 due to artifact exclusions) were square-root transformed and analyzed with the same LMM framework.

Power calculations were informed by effect-size estimates from our previous study using the same heat-pain paradigm and VR Ocean.^3^ In that study, immersion in VR Ocean increased pain-tolerance temperature by 1.025 °C, and the pooled standard deviation across baseline and VR Ocean was approximately 2.53 °C, corresponding to a standardized effect of Cohen's d ≈ 0.41 (small–to–medium).^3^ For the present study, we prespecified a conservative medium effect size of d = 0.50, consistent with Cohen's recommendations and the magnitude observed previously.

To make the calculation reproducible, we used the paired-samples framework appropriate for within-subject designs. The required sample size for detecting d = 0.50 with α = 0.05 and 80% power depends on the within-subject correlation (ρ). Using the standard formula

$$n=\frac{{\left(z_{1-\alpha /2}\;+\;z_{1-\beta }\right)}^{2}\;2\left(1\;-\;\rho \right)}{d^{2}}$$

The required sample varies from n ≈ 63 when ρ = 0.0, to n ≈ 44 when ρ = 0.3, to n ≈ 32 when ρ = 0.5, a range consistent with typical observed correlations in repeated pain-tolerance paradigms.

Because the true within-subject correlation was unknown a priori, and because the study involved seven experimental conditions, covariate adjustment, and expected loss of physiological data in a subset of participants, we selected a conservative recruitment target of 61 participants. This target exceeds the most conservative estimate (ρ ≈ 0) and provides robust power and precision across the planned mixed-effects analyses.

To investigate mechanisms of action, we used multilevel mediation models (MLmed macro v2.30) to test mood, enjoyment, and anxiety as mediators of VR Ocean effects on pain tolerance, pain intensity, and unpleasantness. Condition (VR Ocean vs Baseline) was the predictor (Level 1), mediators and outcomes were VAS scores, and participant ID was entered as the Level 2 cluster. Significance was determined using bootstrapped 95% confidence intervals; indirect effects were deemed significant if the CI excluded zero. Further details are reported in the Supplementary Materials. All statistical significances were set at p < 0.05.

### Ethics

The study was approved by the University of Maryland Institutional Review Board (HP-00069094) and conducted in accordance with the Declaration of Helsinki and Good Clinical Practice. All participants provided written informed consent and were compensated ($50). This study was not a registered clinical trial, as it did not meet the ICMJE definition of a clinical trial.

### Role of the funding source

Funders had no role in study design, data collection, data analysis, interpretation, writing of the report or decision to submit.

## Results

### Demographic and clinical characteristics

Among the 62 participants with TMD, most were females (41–66.1%) with a mean age of 34.7 years (range: 19–55). The majority identified as non-Hispanic or Latino (57–91.9%), and nearly half (30–48.4%) as White. Regarding socioeconomic status, 21.0% (n = 13) had a household income under $20,000, 48.4% (n = 30) were college graduates, and 69.4% (n = 43) were never married (Table 1). Clinically, participants reported moderate pain intensity (49.8 ± 2.8) and interference (31.3 ± 3.6), with 40.4% (n = 25) classified as having high-impact pain. Average pain duration was 125 months (range: 5–504). Psychologically, mean scores were 27.5 on the Beck Depression Inventory and 41.9 on the State-Trait Anxiety Inventory.

### VR effects on heat pain tolerance and physiological arousal

**Fig. 2:**
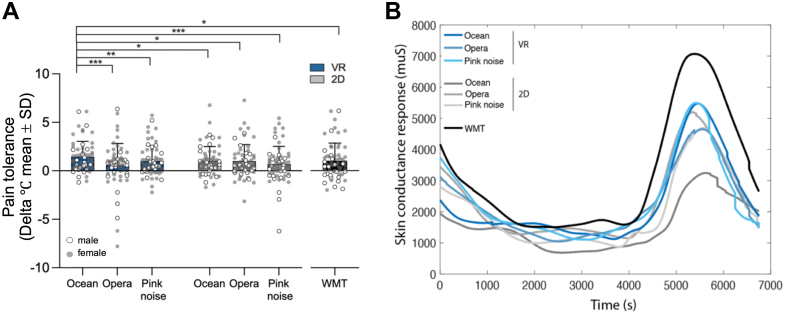
**Heat pain tolerance and skin conductance responses (SCRs) across conditions.** a: Mean increase in heat pain tolerance (°C) relative to baseline across immersive VR (blue shades), non-immersive controls (gray shades), and the working memory task (WMT, black). Error bars represent SD. Asterisks indicate statistically significant differences (∗p < 0.05, ∗∗p < 0.01, ∗∗∗p < 0.001). b: Time course of SCRs (μS) during pain tolerance testing across all conditions. Immersive VR conditions are shown in blue, non-immersive conditions in gray, and the WMT in black. The WMT elicited the highest SCRs, indicating elevated physiological arousal but induced minimal pain tolerance increases.

The VR Ocean condition significantly increased heat pain tolerance compared with all other conditions [F (6, 336) = 4.40, Greenhouse-Geisser p = 0.002; Fig. 2a], including VR Opera, VR Pink Noise, 2D controls, and the Working Memory Task (p < 0.05 for all comparisons; Fig. 2a). In the mixed-effects model, the condition effect was also significant [F (6, 60.01) = 3.989, p = 0.002] where VR Ocean increased pain tolerance compared to every other condition; mean differences ranged from 7.98 to 11.92 units with 95% CIs not crossing 0 (e.g., vs VR Opera: mean difference = −7.984, 95% CI −15.56, −0.402; vs VR Pink Noise mean difference = −9.83, 95% CI −16.86, −2.81; 2D Ocean mean difference = −8.09, 95% CI −15.60, −0.59; 2D Opera mean difference = −10.70, 95% CI −18.81, −2.59; 2D Pink Noise mean difference = −9.77, 95% CI −18.37, −1.17; WMT: mean difference = −11.918, 95% CI −20.887, −2.949). The effect was large (Cohen's d = 1.60, 95% CI = [1.234, 1.974]) and remained after outlier exclusion.

A two-way repeated-measures ANOVA (excluding the Working Memory Task) showed the significant effect of sound type ([F (2, 110) = 8.70, p < 0.001]), but not immersion (VR vs 2D; [F (1, 110) = 0.51, p = 0.478]). Ocean sounds enhanced tolerance more than opera and pink noise; these latter two did not differ. An interaction between immersion and sound type (F (2, 110) = 6.49, p = 0.004) indicated that analgesic effects were driven by emotionally engaging content in VR rather than immersion alone. The corresponding mixed-effects model showed the same pattern where there was a significant main effect of sound type (F (2, 61.003) = 7.752, p = 0.001), but not immersion (F (1, 61.000) = 0.738, p = 0.394), with an interaction between music and sound type (F (2, 61.008) = 3.937, p = 0.025). Within VR, Ocean exceeded Opera (mean difference = 0.88, 95% CI 0.329–1.437, p < 0.001) and Pink noise (mean difference = 0.44, 95% CI 0.112–0.765, p = 0.005).

Findings were independent of sex, race, individual pain sensitivity, clinical pain severity and randomization sequence (all p > 0.47). Exploratory analyses by sex indicated that the effects of VR Ocean on heat pain tolerance were similar in both males and females (main effect of sex (F (1, 68.61) = 1.78, p = 0.187). Similarly, heat pain tolerance did not significantly differ between participants identifying themselves as White and those identifying themselves as other race/ethnicities [F (1, 68.23) = 0.00, p = 0.959]. Also, we found no main effect of the sequence order when set as fixed effect ([F (6, 68.94) = 2.05, p = 0.071], with non-significant randomization order by condition interaction [F (36, 88.33) = 1.35, p = 0.132], Supplementary Figure S2).

SCR showed a main effect of condition (F (6, 132) = 3.78, Greenhouse-Geisser p = 0.020), with the Working Memory Task eliciting greater autonomic arousal than VR Ocean, other VR conditions, and all 2D controls (p < 0.05 for all, Fig. 2b). A follow-up ANCOVA (excluding the Working Memory Task) indicated no significant main effects of immersion [F (1, 46) = 3.09, p = 0.092], sound type [F (2, 46) = 0.18, p = 0.84] or interactions [F (2,46) = 0.12, p = 0.89], indicating that VR Ocean did not differ in physiological arousal from other sound conditions. The mixed effects model showed a main effect of condition on SCR [F (6, 59.2) = 3.45, p = 0.005]. The Working Memory task produced higher arousal than VR Ocean and every other condition for example, vs VR Ocean adjusted mean difference 0.22 μS (95% CI 0.09–0.36, p = 0.001); vs 2D controls 0.18–0.27 μS, all CIs excluding 0. In the mixed-effects model, which excluded the Working Memory Task, neither immersion (F (1, 61.0) = 0.74, p = 0.39) nor music type (F (2, 61.0) = 0.41, p = 0.67) nor their interaction (F (2, 61.0) = 1.12, p = 0.33) was significant. These results suggest that analgesic effects were not explained by sympathetic activation. It is important to note that the results did not differ after multiple imputations to handle the missing data.

### Behavioral self-reported ratings

#### VR effects on pain intensity and unpleasantness

**Fig. 3:**
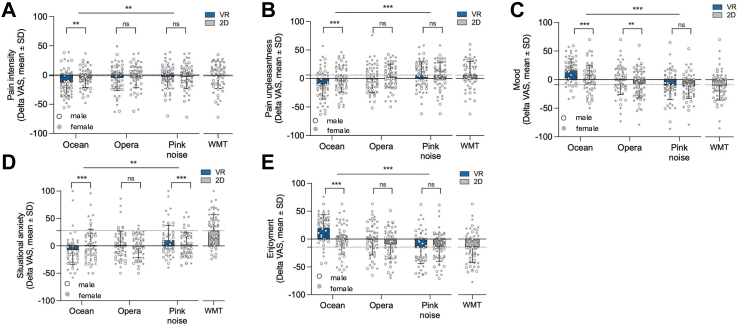
**Subjective pain and affective ratings across immersive and non-immersive conditions.** Delta mean changes from baseline (ΔVAS ± SD) are shown for five outcomes: (a) pain intensity, (b) pain unpleasantness, (c) mood, (d) situational anxiety, and (e) enjoyment. For each outcome, VR conditions (blue bars) consistently produced greater improvements than their 2D counterparts (gray bars). VR Ocean led to the most robust reductions in pain ratings and anxiety, and the greatest improvements in mood and the highest enjoyment. WMT results are included for comparison. Circles represent individual participant scores (gray for female, white for male). Asterisks indicate significance (∗p < 0.05, ∗∗p < 0.01, ∗∗p < *0.001); ns = not significant*.

Condition significantly influenced pain intensity ratings ([F (6,330) = 6.48, p < 0.001; Fig. 3a), with the VR Ocean condition producing the greatest reduction (Cohen's d = 0.60, 95% CI 0.24–0.95), outperforming all other VR, 2D, and distraction conditions (all p < 0.05). A two-way repeated-measures ANOVA (excluding the Working Memory Task) showed the main effects of immersion (F (1, 108) = 5.00, p = 0.030) and sound type (F (2, 108) = 7.22, p = 0.001), and a significant interaction (F (2, 108) = 5.01, p = 0.009). VR Ocean reduced pain more than VR Opera and VR Pink Noise (p < 0.01), whereas no differences were found across sound types in the 2D condition (all p > 0.31). In the mixed effects model, condition significantly influenced pain intensity [F (6, 60.0) = 3.99, p = 0.002]. VR Ocean produced the largest reduction and outperformed every other condition (VR Opera mean difference = −0.88 (95% CI −1.44, −0.33, p < 0.001); VR Pink noise mean difference = −0.44 (95% CI −0.77, −0.11, p = 0.006); 2D Ocean mean difference = −0.63 (95% CI −1.05, −0.21, p = 0.003); 2D Opera mean difference = −0.95 (95% CI −1.50 to −0.40, p < 0.001); 2D Pink noise mean difference = −0.71 (95% CI −1.17 to −0.25, p = 0.002); Working Memory mean difference = −1.12 (95% CI −1.68 to −0.56, p < 0.001).

Excluding the Working Memory Task, there were main effects of Immersion (F (1, 61.0) = 5.21, p = 0.026) and Music type (F (2, 61.0) = 7.10, p = 0.002), and a significant interaction (F (2, 61.0) = 4.95, p = 0.010). Within VR, Ocean had lower intensity than Opera and Pink Noise, while sound type did not differ in 2D (all p > 0.31).

Similarly, condition had a significant effect on pain unpleasantness (F (6, 330) = 8.88, p < 0.001; Fig. 3b), with VR Ocean outperforming most conditions except VR Opera (p = 0.198; all other p < 0.05). The effect size was moderate (Cohen's d = 0.37, 95% CI 0.01–0.72). A two-way ANOVA showed the main effects of immersion (F (1, 108) = 8.16, p = 0.006) and sound type (p < 0.001), and a significant interaction (p < 0.001). Within VR, ocean sound led to significantly greater reductions in unpleasantness than opera and pink noise (p < 0.001); sound type had no effect in 2D. In the mixed effect model, condition also affected unpleasantness [F (6, 59.5) = 4.62, p < 0.001]. VR Ocean exceeded most conditions (VR Opera mean difference = −0.18, 95% CI −0.45, 0.09, p = 0.19); VR Pink noise mean difference = −0.48 (95% CI −0.76, −0.20, p < 0.001); 2D Ocean mean difference = −0.40 (95% CI −0.68, −0.12, p = 0.005); 2D Opera mean difference = −0.58 (95% CI −0.89, −0.27, p < 0.001); 2D Pink noise mean difference = −0.46 (95% CI −0.73,−0.19, p < 0.001); Working Memory Task mean difference = −0.72 (95% CI −1.06, −0.38, p < 0.001). In the model excluding the Working Memory Task, Immersion (F (1, 61.0) = 8.10, p = 0.006) and Music type (F (2, 61.0) = 9.05, p < 0.001) showed main effects, with a significant interaction (F (2, 61.0) = 8.90, p < 0.001). Within VR, Ocean reduced unpleasantness more than Opera (mean difference = −0.62, 95% CI −0.92, −0.32, p < 0.001) and Pink Noise (mean difference = −0.48, 95% CI −0.76, −0.20, p < 0.001); sound type had no effect within 2D.

#### VR effects on mood and situational anxiety

Condition significantly influenced mood ratings (F (6, 330) = 23.08, p < 0.001, Fig. 3c), with VR Ocean producing the greatest improvement (Cohen's d = 0.69, 95% CI 0.32–1.05), outperforming all other VR, 2D, and distraction conditions (all p < 0.001). A two-way repeated-measures ANOVA (excluding the Working Memory Task) showed main effects of immersion (F (1, 108) = 23.13, p < 0.001) and sound type (F (2, 108) = 30.04, p < 0.001), and a significant interaction (F (2, 108) = 8.29, p < 0.001). Within VR, mood improved most with ocean, followed by opera and pink noise (all p < 0.001); in 2D, ocean also outperformed the other sound types (p < 0.009). The mixed effects model Condition was significant [F (6, 60.1) = 9.85, p < 0.001] where again VR Ocean improved mood more than every other condition (VR Opera mean difference = −0.62 (95% CI −0.90, −0.34, p < 0.001), VR Pink noise mean difference = −0.84 (−1.12, −0.56, p < 0.001), 2D Ocean mean difference = −0.41 (−0.60, −0.22, p < 0.001), 2D Opera mean difference = −0.93 (−1.25, −0.61, p < 0.001), 2D Pink noise mean difference = −1.05 (−1.36, −0.74, p < 0.001), Working Memory Task mean difference = −1.20 (−1.55, −0.85, p < 0.001). Excluding the Working Memory task, there were main effects of Immersion (F (1, 61.0) = 12.4, p = 0.001) and Music type (F (2, 61.0) = 16.8, p < 0.001), and a significant interaction (F (2, 61.0) = 6.7, p = 0.002).

Condition also affected situational anxiety (F (6, 330) = 29.01, p < 0.001; Fig. 3d, Cohen's d = 0.232, 95% CI = [−0.121, 0.586]), with VR Ocean reducing anxiety more than VR Opera (p = 0.024), VR Pink Noise (p < 0.001), and the Working Memory Task (p < 0.001). No differences were observed compared with 2D control conditions (all p > 0.14). A two-way ANOVA revealed a main effect of sound type (F (2, 108) = 7.98, p < 0.001), but not immersion ([F (1, 108) = 0.31, p = 0.582]), and a significant interaction (F (2, 108) = 13.06, p < 0.001). In VR, ocean reduced anxiety more than both opera (p = 0.005) and pink noise (p < 0.001); no significant differences emerged in the 2D condition (all p > 0.70). Condition was also significant in the mixed-effects model, [F (6, 59.7) = 5.21, p < 0.001]. VR Ocean reduced anxiety considerably (VR Opera mean difference = −0.21, 95% CI −0.38, −0.04, p = 0.014), VR Pink noise mean difference = −0.35, 95% CI −0.51, −0.19, p < 0.001), and Working Memory mean difference = −0.48, 95% CI −0.67, −0.29, p < 0.001). No significant difference was observed in the 2D controls. In the model excluding the Working Memory Task, there was a main effect of Music type (F (2, 61.0) = 7.9, p = 0.001), but not Immersion (F (1, 61.0) = 0.31, p = 0.58), and a significant interaction (F (2, 61.0) = 12.6, p < 0.001). Within VR, Ocean < Opera (p = 0.005) and Ocean < Pink (p < 0.001) for anxiety; within 2D, sound types did not differ (all p > 0.70).

#### VR effects on enjoyment

Condition significantly affected enjoyment ratings (F (6, 330) = 22.50, p < 0.001; Fig. 3e), with VR Ocean rated as more enjoyable than all other conditions (Cohen's d = 0.65, 95% CI 0.28–1.02; all p < 0.001). A two-way repeated-measures ANOVA indicated main effects of immersion (F (1, 108) = 25.24, p < 0.001) and sound type (F (2, 108) = 29.90, p < 0.001), as well as a significant interaction (F (2, 108) = 14.43, p < 0.001). Enjoyment was higher in VR than 2D, and ocean sound was rated more enjoyable than opera and pink noise across formats. Within VR, ocean elicited the highest enjoyment (p < 0.001); within 2D, ocean was rated higher than pink noise (p = 0.002) but not opera (p = 0.810). Mixed-effects modeling confirmed a significant condition effect (F (6, 60.2) = 10.9, p < 0.001), with VR Ocean outperforming all other conditions. Interestingly, 69.9% of participants identified VR Ocean as the condition providing the greatest sense of pain control, independent of depression, anxiety, mood regulation, and music reward sensitivity (all p > 0.41).

#### Mood and enjoyment mediation effects

**Fig. 4:**
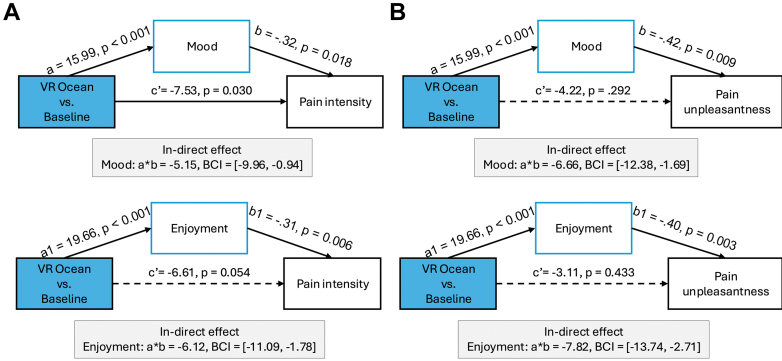
**Mood and enjoyment mediate the effects of VR Ocean on pain outcomes.** Mediation analyses indicated that mood (a) and enjoyment (b) significantly mediated the effect of VR Ocean on both pain intensity and unpleasantness. These findings suggest that emotional and affective engagement play a key mechanistic role in VR-induced analgesia.

Mood and enjoyment significantly mediated the analgesic effects of the VR Ocean condition on both pain intensity and unpleasantness. For pain intensity, mood (ab = −5.15, 95% BCI –9.96 to −0.94) and enjoyment (ab = −6.12, 95% BCI –11.09 to −1.78) each contributed to reduced pain ratings (Fig. 4a), whereas situational anxiety did not (ab = −1.37, 95% BCI –3.98 to 0.21). A similar pattern was observed for pain unpleasantness, with mood (ab = −6.66, 95% BCI –12.38 to −1.69) and enjoyment (ab = −7.82, 95% BCI –13.74 to −2.71) emerging as significant mediators (Fig. 4b), and no significant mediation via anxiety (ab = −0.91, 95% BCI –3.61 to 1.00).

In contrast, no significant indirect effects were found for heat pain tolerance. Mediation paths through mood (ab = 0.12, 95% BCI –0.19 to 0.46), enjoyment (ab = 0.05, 95% BCI –0.27 to 0.40), or situational anxiety (ab = −0.08, 95% BCI –0.26 to 0.03) were small and non-significant, suggesting that subjective improvements in affect do not account for changes in tolerance thresholds.

#### Comparison of VR effects with healthy controls

Using data from a previously published study,^3^ we compared VR effects across five conditions between individuals with chronic pain (TMD) and healthy controls (HC) using repeated-measures ANCOVA. No significant group differences were observed in pain unpleasantness (F (4, 392) = 0.28, Greenhouse-Geisser p = 0.890), mood (F (4, 392) = 0.58, Greenhouse-Geisser p = 0.652), or heat pain tolerance (F (4, 392) = 1.15, Greenhouse-Geisser p = 0.329), indicating comparable effects of VR Ocean across groups (Supplementary Figure S2). A significant group-by-condition interaction was found for situational anxiety (F (4, 392) = 3.37, Greenhouse-Geisser p = 0.013), with TMD participants showing a trend toward higher anxiety during the Working Memory Task (p = 0.062) but similar reductions to HC in both VR and 2D conditions (all p > 0.26). Regarding safety, none of the participants despite having orofacial pain reported discomfort while wearing the VR headset.

## Discussion

We investigated how audiovisual content affects VR's impact on pain tolerance using a within-subject design with three immersive VR conditions (relaxing ambient sound, arousing opera, and neutral pink noise) each paired with a 2D control. Findings showed that VR with ocean ambient sound significantly increased pain tolerance compared to all other conditions, including VR opera, pink noise, 2D controls, and the working memory task (used as a proxy for non-specific effects of distraction). Ocean sound also produced the largest reductions in pain unpleasantness and anxiety, and improvements in mood and users' enjoyment. These results suggest that analgesic effects depend more on the emotional and sensory qualities of VR content than immersion alone. In particular, the combination of nature-based visuals and calming ambient sound produced the most consistent improvements in pain tolerance, affect, and engagement. While these findings highlight the potential of immersive, affectively positive VR environments, generalizations to other sound genres or auditory contexts should be made with caution, as the present study examined only one type of ambient sound within a nature-based setting. Opera effects varied likely due to individual differences, while pink noise was less engaging. The working memory task increased physiological arousal but was less effective for pain relief.

Immersion enhanced the effect of emotionally engaging content rather than driving analgesia itself. Mood significantly mediated VR Ocean's impact on pain intensity and unpleasantness, highlighting positive mood as a key mechanism. Overall, immersive, calming audiovisual environments that integrate nature elements and low-arousal sound may represent promising non-pharmacological options for chronic pain management, though future research should systematically test different sound styles and emotional tones to determine the boundaries of these effects (Supplementary Figure S4).

SCR levels are greater during painful conditions, as compared to non-painful conditions.^11^, ^12^, ^13^ Using the method of limits,^30^ participants reported the highest tolerable heat intensity during the Ocean condition with ambient sound. This increase in thermal pain tolerance was independent from arousal observed during the working memory task, consistent with our prior findings in healthy controls.^3^

Behaviorally, our findings are consistent with the literature indicating that both nature and sound, especially when designed or selected to induce calm and positive affect,^9^ can reduce pain perception and modulate physiological responses. For example, positive affect has been associated with health benefits including immune and neuroendocrine systems.^31^ Nature environments such as a virtual coastal walk were reported to reduce experienced and recollected pain.^32^ Surgical patients with a view of trees required less pain medication, received more favorable notes from staff, and were discharged sooner than those facing a brick wall.^33^ In healthy controls, nature exposure was associated with a reduced response to a neurological signature of pain (the NPS,^34^) linked to nociception-related brain processes.^35^ In chronic pain populations, simulated nature such as videos of forests or beaches has been associated with reduced pain intensity, anxiety, and blood pressure, even during brief exposures.^36^ Importantly, we found a dissociation between subjective pain ratings and pain tolerance which suggests that distinct mechanisms underlie these outcomes. While mood and enjoyment seem to modulate the *experience* of pain, tolerance may depend more on sensorial control processes that are less sensitive to affective modulation. This distinction highlights separate affective and sensorial pathways through which VR can influence pain.

Sound, particularly slow-tempo, consonant, and low-arousal sounds and music, has long been associated with analgesia, reduced cortisol and improved mood.^6^^,^^37^ Importantly, individual preference and context matter: listening to preferred sounds and music genres enhances pain tolerance, regardless of the genre itself.^38^ This may explain why ambient sound paired with ocean visuals consistently ranked more enjoyable and was more effective than opera (which, despite being immersive, can elicit mixed or emotionally intense responses depending on the listener).

To extend our prior research, we introduced a third immersive condition featuring pink noise paired with repetitive visual stimuli. Alongside the nature-based VR Ambient sound and the emotionally rich VR Opera environments, this addition enabled a more comprehensive assessment of how varied auditory and visual inputs contribute to VR's analgesic effects.^39^ Pink noise lacks emotional or musical structure. While sometimes beneficial in sleep research,^40^ its repetitive and flat auditory profile is less likely to evoke emotional responses or support attentional engagement factors. In fact, while both relaxing sound/music and an individual's favorite music reduced muscular effort during spontaneous awake bruxism episodes in individuals with TMD, pink noise had no such effect.^41^ Its inclusion in the present study strengthens the conclusion that it is not immersion per se, but the quality and emotional aspect of the immersive content that is critical to its analgesic effect.

VR Ocean produced comparable benefits in individuals with chronic pain (e.g., TMD) and healthy controls,^3^ with no significant group differences in pain unpleasantness, mood, or heat pain tolerance. These findings suggest that the analgesic and affective benefits of immersive VR are preserved in clinical populations, supporting its broader applicability in chronic pain management.

With regards to immersion as an amplification of the effects of other stimuli, this finding is in line with our recent results.^42^ We recently conducted a randomized crossover trial involving individuals with chronic TMD, comparing a five-day, self-administered VR intervention to both an MP3-based audio program and a non-intervention control.^42^ Using ecological momentary assessment and PROMIS outcomes, we found that the VR intervention led to greater reductions in pain intensity, pain interference, and anxiety, along with improvements in mood and sleep. Because the VR and MP3 conditions were matched for therapeutic content (mindfulness, breathing, and acceptance-based practices) our previous study suggests the added value of immersive VR beyond the therapeutic content itself.

### Limitations

Our study focused on sound-based immersive content, without broader therapeutic modules. Future studies should compare patient-centered, sound-based VR with established pain management programs (e.g.,^43^) to better understand their relative and additive benefits. A key limitation of our current study is that the VR conditions differed in both visual and auditory features, making it difficult to isolate the specific contribution of each modality. As such, the enhanced effects observed in the VR Ocean condition may be attributed to either the calming sound, the nature-based visuals, or the combination of both. In this sense, one could also interpret the study as a comparison of distinct visual VR environments rather than auditory effects alone. While a fully orthogonal manipulation of auditory and visual components would better isolate their effects,^5^ this study prioritized ecological validity and feasibility. Although perceived immersion ratings were not collected, this represents an important limitation. We incorporated self-report measures of subjective engagement in a recent study^5^ and found that it contributes to analgesic outcomes. In addition, the relatively small and geographically homogeneous sample limits the generalizability of our findings. To avoid model overfitting, we did not include multiple covariates in the present analyses; future work with larger and more diverse samples should employ multivariate or mixed-effects approaches to account for individual-level moderators. Examining intra-individual response patterns to identify subgroups of participants who are more or less responsive could further elucidate variability in treatment effects. Although a 6–8 min washout period was included between conditions, we cannot exclude residual experiential or expectancy-related carryover effects inherent to immersive within-participant designs, nor the possibility of demand characteristics arising from repeated comparative judgments. Finally, although we reported results from a 5-week follow-up in the same population,^42^ longitudinal studies assessing whether the analgesic effects persist after repeated sessions or over extended periods remain an important direction for future research.

Our findings support the potential of pleasant sound- and scenery-based VR as a promising tool for pain modulation. Recent technological advancements have made VR more affordable, portable, and user-friendly, increasing its feasibility in both clinical and home settings. Positive public perception, early cost-effectiveness analyses, and policy advocacy for insurance coverage further support its integration into standard care. Understanding the optimal characteristics of VR environments is essential for designing effective interventions for individuals with chronic pain.

## Contributors

Study design: L. Colloca, N. Raghuraman, S. Murthi, A. Varshney and G. Colloca. Context design (immersive VR OPERA): B. Brawn, A. Varshney, C. Kier Data collection: N. Raghuraman. Data Access and analysis: N Raghuraman and Y. Wang. Draft: N. Raghuraman, R. Shafir, G. Colloca, Y. Wang and L. Colloca. Final version of the manuscript: L. Colloca, N. Raghuraman, G. Colloca, B. Brawn. All authors approved the final version of the manuscript.

## Data sharing statement

All deidentified participant data and dictionary will be made available upon request to the corresponding authors.

## AI used statement

AI-assisted tools were used solely to improve language and readability. All scientific content, analyses, and interpretations were generated by the authors.

## Declaration of interests

All authors have no conflict of interest to declare.

## Appendix A Supplementary data

Supplementary Material

Supplementary captions

## References

[1] Malloy K.M., Milling L.S. (2010). The effectiveness of virtual reality distraction for pain reduction: a systematic review. Clin Psychol Rev.

[2] Gupta A., Scott K., Dukewich M. (2018). Innovative technology using virtual reality in the treatment of pain: does it reduce pain via distraction, or is there more to it?. Pain Med.

[3] Colloca L., Raghuraman N., Wang Y. (2020). Virtual reality: physiological and behavioral mechanisms to increase individual pain tolerance limits. Pain.

[4] Morris N.A., Wang Y., Felix R.B. (2023). Adjunctive virtual reality pain relief after traumatic injury: a proof-of-concept within-person randomized trial. Pain.

[5] Shafir R., Watson L., Felix R.B. (2025). Factors influencing the hypoalgesic effects of virtual reality. Pain.

[6] Yi W., Palmer C., Serian A., Roy M. (2025). Individualizing musical tempo to spontaneous rates maximizes music-induced hypoalgesia. Pain.

[7] Tesarz J., Herpel C., Meischner M. (2024). Effects of virtual reality on psychophysical measures of pain: superiority to imagination and nonimmersive conditions. Pain.

[8] Blood A.J., Zatorre R.J. (2001). Intensely pleasurable responses to music correlate with activity in brain regions implicated in reward and emotion. Proc Natl Acad Sci U S A.

[9] Fredrickson B., Cohn M., Lewis M., Haviland-Jones J., Barrett L. (2016).

[10] National Academies of Sciences E, Medicine (2020).

[11] Dube A.A., Duquette M., Roy M., Lepore F., Duncan G., Rainville P. (2009). Brain activity associated with the electrodermal reactivity to acute heat pain. Neuroimage.

[12] Eriksson M., Storm H., Fremming A., Schollin J. (2008). Skin conductance compared to a combined behavioural and physiological pain measure in newborn infants. Acta Paediatr.

[13] Schestatsky P., Valls-Solé J., Costa J., León L., Veciana M., Chaves M.L. (2007). Skin autonomic reactivity to thermoalgesic stimuli. Clin Auton Res.

[14] Forte G., Troisi G., Pazzaglia M., Pascalis V.D., Casagrande M. (2022). Heart rate variability and pain: a systematic review. Brain Sci.

[15] Schiffman E., Ohrbach R., Truelove E. (2014). Diagnostic criteria for temporomandibular disorders (DC/TMD) for clinical and research applications: recommendations of the international RDC/TMD consortium network∗ and orofacial pain special interest groupdagger. J Oral Facial Pain Headache.

[16] Zakrzewska J.M. (2013). Differential diagnosis of facial pain and guidelines for management. Br J Anaesth.

[17] Dixon D., Pollard B., Johnston M. (2007). What does the chronic pain grade questionnaire measure?. Pain.

[18] Von Korff M., Dworkin S.F., Le Resche L. (1990). Graded chronic pain status: an epidemiologic evaluation. Pain.

[19] Dworkin S.F., Huggins K.H., Wilson L. (2002). A randomized clinical trial using research diagnostic criteria for temporomandibular disorders-axis II to target clinic cases for a tailored self-care TMD treatment program. J Orofac Pain.

[20] Ohrbach R., Larsson P., List T. (2008). The jaw functional limitation scale: development, reliability, and validity of 8-item and 20-item versions. J Orofac Pain.

[21] Ohrbach R., Markiewicz M.R., McCall W.D. (2008). Waking-state oral parafunctional behaviors: specificity and validity as assessed by electromyography. Eur J Oral Sci.

[22] Maixner W., Fillingim R.B., Williams D.A., Smith S.B., Slade G.D. (2016). Overlapping chronic pain conditions: implications for diagnosis and classification. J Pain.

[23] Beck A.T., Steer R.A., Carbin M.G. (1988). Psychometric properties of the beck depression inventory: twenty-five years of evaluation. Clin Psychol Rev.

[24] Barker H.R., Wadsworth A.P., Wilson W. (1976). Factor structure of the State-Trait Anxiety Inventory in a nonstressful situation. J Clin Psychol.

[25] Wadsworth A.P., Barker H.R., Barker B.M. (1976). Factor structure of the State-Trait Anxiety Inventory under conditions of variable stress. J Clin Psychol.

[26] Tran T.D., Tran T., Fisher J. (2013). Validation of the depression anxiety stress scales (DASS) 21 as a screening instrument for depression and anxiety in a rural community-based cohort of northern Vietnamese women. BMC Psychiatry.

[27] Gross J.J., John O.P. (2003). Individual differences in two emotion regulation processes: implications for affect, relationships, and well-being. J Pers Soc Psychol.

[28] Mas-Herrero E., Marco-Pallares J., Lorenzo-Seva U., Zatorre R.J., Rodriguez-Fornells A. (2013). Individual differences in music reward experiences. Music Percept.

[29] Gentile D. (2009). Pathological video-game use among youth ages 8 to 18: a national study. Psychol Sci.

[30] Fruhstorfer H., Lindblom U., Schmidt W.C. (1976). Method for quantitative estimation of thermal thresholds in patients. J Neurol Neurosurg Psychiatry.

[31] Segerstrom S.C., Sephton S.E. (2010). Optimistic expectancies and cell-mediated immunity: the role of positive affect. Psychol Sci.

[32] Tanja-Dijkstra K., Pahl S., White M.P. (2018). The soothing sea: a virtual coastal walk can reduce experienced and recollected pain. Environ Behav.

[33] Ulrich R.S. (1984). View through a window may influence recovery from surgery. Science.

[34] Wager T.D., Atlas L.Y., Lindquist M.A., Roy M., Woo C.-W., Kross E. (2013). An fMRI-based neurologic signature of physical pain. N Engl J Med.

[35] Steininger M.O., White M.P., Lengersdorff L. (2025). Nature exposure induces analgesic effects by acting on nociception-related neural processing. Nat Commun.

[36] Lee M.J., Pradeep A., Lobner K., Badaki-Makun O. (2023). The effect of nature exposure on pain experience and quality of life in patients with chronic pain: a systematic review and meta-analysis protocol. PLoS One.

[37] Roy M., Peretz I., Rainville P. (2008). Emotional valence contributes to music-induced analgesia. Pain.

[38] Van der Valk Bouman E.S., Becker A.S., Schaap J. (2024). The impact of different music genres on pain tolerance: emphasizing the significance of individual music genre preferences. Sci Rep.

[39] Honzel E., Murthi S., Brawn-Cinani B. (2019). Virtual reality, music, and pain: developing the premise for an interdisciplinary approach to pain management. Pain.

[40] Capezuti E., Pain K., Alamag E., Chen X., Philibert V., Krieger A.C. (2022). Systematic review: auditory stimulation and sleep. J Clin Sleep Med.

[41] Imbriglio T.V., Moayedi M., Freeman B.V., Tenenbaum H.C., Thaut M., Cioffi I. (2020). Music modulates awake bruxism in chronic painful temporomandibular disorders. Headache.

[42] Colloca L., Han A., Massalee R. (2025). Telehealth virtual reality intervention reduces chronic pain in a randomized crossover study. NPJ Digit Med.

[43] Maddox T., Oldstone L., Sparks C.Y. (2023). In-Home virtual reality program for chronic lower back pain: a randomized sham-controlled effectiveness trial in a clinically severe and diverse sample. Mayo Clin Proc Digit Health.

