# Supplementary material for: A critical role of affective content in the analgesic effect of virtual reality: a cross-sectional within-subject study
Source: Lancet Reg Health Am. 2026 Feb 16;55:101385. doi: 10.1016/j.lana.2026.101385 (PMC12930066; doi:10.1016/j.lana.2026.101385)
Supplement: Supplementary Material [file mmc1.docx]

**Supplementary Materials**

**A critical role of affective content in the analgesic effect of virtual reality: a cross-sectional within-subject study**

Nandini Raghuraman, PhD^1,2^, Roni Shafir, PhD^1,2^, GianCarlo Colloca MS, MM^3^, Craig Kier, MM^4^, Barbara Brawn, MS^5^, Amitabh Varshney, PhD^6^, Sarah Murthi, MD^7^, Yang Wang, PhD^1,2,8^, Luana Colloca, MD, PhD^1,2,8,*^

1. Department of Pain and Translational Symptom Science, School of Nursing, University of Maryland, Baltimore, Maryland, USA
2. Placebo Beyond Opinions Center, School of Nursing, University of Maryland, Baltimore, Maryland, USA
3. Towson University, Information Technology Program, Towson, Maryland, USA
4. School of Music, University of Maryland College Park, Maryland, USA
5. Department of Computer Science, Institute for Advanced Computer Studies, University of Maryland, College Park, Maryland, USA
6. Department of Computer Science, University of Maryland, College Park, Maryland, USA
7. Division of Trauma and Critical Care, R Adams Cowley Shock Trauma Center, University of Maryland, School of Medicine, Baltimore, Maryland, USA
8. Center to Advance Chronic Pain Research, University of Maryland, Baltimore, Maryland, USA

*Correspondence to: Luana Colloca, MD, PhD, MS - 655 W. Lombard Street Room 733, 21201 Baltimore, MD; Phone: +1 410-706-8244; Fax: +1 410-706-5427; email: [colloca@umaryland.edu](mailto:colloca@umaryland.edu) or [nraghuraman@umaryland.edu](mailto:nraghuraman@umaryland.edu)

**Methods**

**Recruitment strategy:** Our advertising strategy utilized various channels, including social media, advertising in local newspapers, online platforms and participant recruitment websites. We engaged with local healthcare dentists for referrals, and we attended academic fairs, music festivals, and health fairs. Moreover, we established an IRB-approved online social media presence to enhance our outreach efforts. We adopted a "word of mouth" strategy whereby participants referred to other TMD. The Consort below shows that of the 78 individuals screened, 68 were enrolled (25 men, 43 women). Two participants were lost to follow-up, and four were excluded from the primary outcome analysis due to missing data, resulting in 62 participants (21 men, 41 women) included in the primary outcome analysis. For the secondary outcomes, data from 25 participants were excluded due to poor-quality physiological recordings, with final analytic sample of 41 participants (13 men, 28 women). A total of 62 participants (21 men, 41 women) were included for the behavioral secondary outcomes (pain intensity and unpleasantness, situational anxiety, mood, and enjoyment).

Screened

N = 78 (30 M / 48 F)

**Suppl Figure 1.**

Enrolled

n = 68 (25 M / 43 F)

Primary outcome analysis

n = 62 (21 M / 41 F)

Secondary outcomes (behavioral)

n = 62 (21 M / 41 F)

Secondary outcome (physiological)

n = 41 (13 M / 28 F)

Completed all procedures

n = 66 (24 M / 42 F)

**TMD Diagnostic Procedure:** Those TMD participants enrolled in the study were screened no more than three months before the start of the study. The diagnostic criteria for TMD were confirmed with an examination conducted by an independently trained, calibrated examiner according to the Axis I Diagnostic Criteria for TMD (DC/TMD) at the Brotman Facial Pain Clinic, School of Dentistry University of Maryland. A dental clinician at the Brotman Facial Pain Clinic of the University of Maryland School of Dentistry (UMSOD) confirmed the self-reported history of at least three months of jaw, head, or facial pain and to determine the phenotypic classification and eligibility of their orofacial pain condition. Myalgia and/or arthralgia in one or both temporomandibular joints contribute to TMD-related discomfort. Included in the study were participants who met the DC/TMD ^1,2^: a. Pain in the jaw, temple, in the ear, or in front of the ear; and b. Pain modified by jaw movement, function, or parafunction; and c. History of pain in the jaw, temple, ear, or in front of the ear within the previous 30 days (about 4 and a half weeks); and d. a. Confirmation and duplication of pain location(s) in the temporalis, masseter, or other masticatory muscle(s); and/or b. Confirmation and duplication of pain in one or more temporomandibular joints.

**Additional Clinical Pain Assessments:** Axis II evaluation via the Graded Chronic Pain Scale ^3^. Chronic pain severity and disability were assessed and used as a secondary outcome in the sub-group analysis. According to Von Korff et al. ^4^, participants were classified into five hierarchical categories based on pain intensity and disability levels: Grade 1 (low pain intensity and low disability), Grade 2a (high pain intensity without disability), Grade 2b (high pain intensity with low disability), Grade 3 (moderately limiting pain), and Grade 4 (severely limiting pain). Following Dworkin et al. ^5^, Grades 1 and 2a were grouped as low-impact pain, while Grades 2b through 4 were categorized as high-impact pain. TMD often is accompanied by other chronic pain overlapping comorbidities (COPCs)6 and participants reported them. Jaw Function Limitation Scale ^7^, and the Oral Behaviors Checklist for parafunctional behaviors ^8^ were also assessed.

**Other questionnaires:** After the experimental session, participants were asked to complete a series of questionnaires. To measure depression and anxiety, we used Beck’s Depression Inventory (BDI ^9^), the State-Trait Anxiety Inventory-Trait (STAI-Trait ^11,12^), and the Depression Anxiety Stress Scale (DASS ^10^). We also utilized the Emotion Regulation Questionnaire (ERQ ^14^) to measure participants’ emotion regulation tendencies. In addition, we assessed individual differences in music reward sensitivity using the Barcelona Music Reward Questionnaire (BMRQ) ^15^, which captures higher-order pleasure responses to music across five domains: musical seeking, emotion evocation, mood regulation, social reward, and sensory-motor engagement. The Gaming Addiction Survey ^16^ was administered to assess any pre-existing video game addiction. Lastly, we assessed the users’ experience by asking participants to indicate which condition was the most effective in reducing how unpleasant and emotionally upsetting the pain stimuli felt and to what degree they felt this condition allowed them to control how unpleasant and emotionally upsetting the pain stimuli felt.

**Procedures**

All participants spoke and understood English. Exclusion criteria included having facial trauma within the past 6 weeks, a history of severe facial trauma within the past 2-3 months, degenerative neuromuscular diseases, cervical pain (i.e. stenosis or radiculopathy), cardiovascular, neurological, kidney, or liver diseases, pulmonary abnormalities, diffuse cancer within the past three years, color-blindness, uncorrected impaired hearing, current pregnancy or breastfeeding, a lifetime history of alcohol or drug dependence, alcohol or drug abuse in the past year, a severe psychiatric condition requiring medication or hospitalization within the past three years, and vertigo.

After signing the informed consent form, participants began the experimental session, which took place at the UMSON clinical research space and lasted approximately two hours. First, heart rate and blood pressure were measured to establish baseline health status. Additionally, height and weight were measured to calculate the Body Mass Index (Table 1). These measurements were assessed using Heart Rate Welch Allyn Connex ProBP 3400 Digital Blood Pressure Device (blood pressure and heart rate) and the Cardinal Detecto solo 550 lb. Digital Scale with Mechanical In-Line Height Rod was used to measure weight and height. The BMI was calculated using the National Heart Lung and Blood Institute's Online BMI calculator (<https://www.nhlbi.nih.gov/health/educational/lose_wt/BMI/bmi-m.htm>).

For the GSR measurement, two electrodes were positioned on the bottom of the left palm. Data were recorded using the BrainAmp ExG amplifier and BrainVision Recorder (Brain Products GmbH, Munich, Germany). GSR was recorded 4 seconds after the cessation of each pain stimulus to assess autonomic sympathetic responses for each experimental condition.

SCR data were recorded using the Brain Vision Recorder system (Brain Products GmbH, Munich, Germany). Data analysis was performed with the LedaLab V3.4.6c software package for MATLAB ^17^. The raw data were initially downsampled to 250 Hz^18^ and smoothed using a moving average filter with a 1000-sample window. Subsequently, a low-pass filter with a 2 Hz cut-off was applied to remove high-frequency noise ^17^. Following pre-processing, continuous decomposition analysis (CDA) was conducted to separate the signal into tonic and phasic components. The tonic component, also referred to as the skin conductance level (SCL), reflects the baseline autonomic activity. The phasic component, or skin conductance response (SCR), captures stimulus-related responses. For each trial, SCRs were extracted from a window spanning 1 second after heat onset to 4 seconds after heat offset, accounting for delayed autonomic responses ^18^. The area under the curve (AUC) of the SCR was calculated separately for each of the seven experimental conditions.

**Pain Sensitivity Assessment:** Individual pain measures for warmth, heat pain threshold, medium pain, and maximum pain tolerance were assessed to evaluate personalized pain sensitivity, utilizing a version of the quantitative sensory testing (QST)^19^ (note that cold pain sensitivity was not assessed). This investigation was carried out using the limits procedure ^20^. A 3x3 cm thermode was used along with the PATHWAY (Pain and Sensory Evaluation System) device from Medoc Advanced Medical Systems, based in Ramat Yishai, Israel. To control for potential regional variations in pain sensitivity, thermal stimuli were applied to the same location on participants’ dominant forearm. The thermode delivered heat stimuli ranging from 32 °C to 50 °C. At the beginning of the pain sensitivity test, participants were given a control (stop) button and instructed to press it as soon as they first felt a warm thermal sensation (i.e., warmth). They were instructed to press the stop button when they felt even the slightest heat pain. Then, participants were asked to press the stop button at three distinct points: when they first felt minimal heat pain (i.e., heat pain threshold), when they reached a medium level of heat pain (i.e., medium pain), and finally when they experienced their highest tolerable level of pain (i.e., maximum pain tolerance). The levels of warmth, heat pain threshold, medium pain, and maximum pain tolerance were each measured three times. Additionally, each time, participants were asked to rate their pain verbally on a scale from 0 to 100, with 0 representing no pain and 100 representing the highest level of pain they could tolerate. The same Pain Sensitivity Assessment was later repeated seven times during each experimental condition.

**Conditions and timeline**

After the familiarization, the TMD participants underwent a within-subjects design, with the experimental conditions being counterbalanced. The experimental conditions included:

***Immersive VR Ocean*:** The VR ambient music condition used the Blue Season 1 (Wevr, Venice, California, USA) to immerse participants in the ‘Reef Migration’ episode. This VR episode placed participants deep in the ocean, surrounded by fish, jellyfish, and other aquatic life, accompanied by nature music as auditory stimulation.

***Immersive VR Opera*:** The VR Opera condition was produced at the University of Maryland College Park, Maryland Blended Reality Center. This episode featured segments from the opera *La Clemenza di Tito*, K. 621. The immersive VR experience transported participants into a virtual theatre, where they experienced 360-degree video and audio immersion of the opera performance.

***Immersive VR Pink noise*:** The VR Pink Noise condition included being immersed in a repetitive pink bubble visual stimuli environment (adapted from [https://youtu.be/ZxtimhT-ff4?si=svYe27o5jx5_9XIK](https://youtu.be/ZXtimhT-ff4?si=svYe27o5jx5_9XIK)), accompanied by pink noise. Pink noise, with its deeper, balanced sound and enhanced low frequencies compared to white noise, mimics natural sounds like ocean waves and waterfalls. It is commonly used for relaxation, focus, and masking background noise.

***Non-immersive (control) Ocean*:** To eliminate the immersive component, the Control VR Ambient music condition involved a non-immersive version of the Blue Season 1 (Wevr, Venice, California, USA), Reef Migration episode, presented on a tablet (iPad Pro, 10.5-inch) with headphones (Audio-Technica ATH-M20x Professional Studio Monitor Headphones Deluxe Bundle).

***Non-immersive (control) Opera*:** Similarly, the Control VR Opera condition featured the same opera performance, *La Clemenza di Tito*, delivered through the tablet and headphones.

***Non-immersive (control) Pink noise*:** The same setup was used for the Control VR Pink Noise condition, where pink bubbles fluctuated on the 2D tablet screen with pink noise played through the headphones.

***2-back working memory task:*** The task was adapted to control for the effect of distraction on pain, as previously described and implemented ^21^. It involved displaying 90 capitalized letters one at a time in a pseudorandom sequence on a 22-inch 2D monitor. Participants were asked to determine whether the displayed letter differed from the one shown two steps prior. The letters, presented in Arial font on a white background, were displayed for 500 ms, followed by a 1500 ms blank screen. Of the 90 trials, 30 were target trials where the current letter **matched** the letter presented two trials earlier**,** and 60 were non-target trials where the current letter did not **match** the letter presented two trials earlier. Participants used the Celeritas® Fiber Optic Response System, pressing the thumb button for target trials and the index button for non-target trials.

These three VR conditions were delivered using a workstation CPU with an NVIDIA K6000 GPU (Alienware 17 R4 – Alienware Miami, Florida, USA) and an HTC Vive Pro headset (HTC Xindian, New Taipei, Taiwan). Each participant wore the headset and experienced the VR episodes while the experimenter watched the VR episodes via an LED display panel. Participants were told that immersion in the VR may cause a sense of being in a closed environment and, rarely, nausea and that if they had experienced any discomfort, the VR would have been removed quickly. No side effects were reported for any VR conditions during the experimental sessions, and none of the study participants withdrew from the VR experience. When asked at the end of the experiment, participants referred to the VR as an experience that they would like to re-try.

**Primary Outcome.** Heat pain tolerance levels were calculated by averaging the temperature (°C) reached across the four maximum pain tolerance measurements.

**Secondary Outcomes.** Participants completed the Pain Sensitivity Assessment during each experimental condition: VR Ocean, VR Opera, VR Pink Noise, Control VR Ocean, Control VR Opera, Control VR Pink Noise, and Working Memory Task. 100-anchorated VAS scales were used to assess self-reported pain experience following each experimental condition. Specifically, we measured Pain Intensity (“Please rate your overall pain intensity during the session”), Pain Unpleasantness (“Please rate your overall pain unpleasantness during the session”), Anxiety (“Please rate your anxiety during the session), Mood (“Please rate your mood level during the session”), and Enjoyment (“Please rate how much you liked the session”). The anchors were set, respectively, with 0 being “no pain at all”, “not unpleasant at all”, “not anxious at all”, “extremely bad mood”, and “not enjoyable at all” and 100 being “maximum tolerable pain”, “very unpleasant”, “very anxious”, “extremely good mood”, and “very enjoyable”. The distance from the zero to the end anchor (i.e., 100) was electronically recorded. VAS ratings were acquired using Eprime v2 (Psychology Software Tools, Sharpsburg, PA, USA) and participants operated a Celeritas Fiber Optic Response System (Sharpsburg, PA, USA), using their index and middle fingers to move a slider on the VAS scale. Eprime v2 (Psychology Software Tools Inc, Sharpsburg, USA) and Matlab were used to extract the data.

**Sociodemographic Assessments:** Self-reported ethnicity and race were captured using the standardized categories mandated by NIH to ensure consistency and comparability across studies. Ethnicity was reported first, with participants categorized as **Hispanic or Latino or Not Hispanic or Latino**. Race was reported using five categories: **American Indian or Alaska Native, Asian, Black or African American, Native Hawaiian or Other Pacific Islander**, and **White.** Participants were allowed to select one or more races, and those who chose more than one category were grouped as “mixed race.” This standardized approach ensured the accurate capturing of diversity. A demographic questionnaire determined the age, education, annual income, and occupation.

**Statistical Analysis**

An independent investigator not involved in study design and data collection analyzed the data. Primary and secondary outcomes were analyzed using a combination of repeated-measures ANOVAs and multivariate models. We initially conducted one-way repeated measures ANOVA across all seven experimental conditions to assess overall effects. Following this, we omitted the working memory condition and performed a two-way factorial repeated measures ANOVA on the remaining six groups, with auditory content and visual immersion as within-subject factors.

To examine the role of demographics on primary and secondary outcomes, we conducted multivariate linear regression analyses using age, sex (men vs. women), and self-reported race as independent variables, and VR-induced changes in pain tolerance as the dependent variable. Where applicable, post-hoc comparisons were adjusted using Bonferroni correction.

We computed effect sizes for each VR and baseline condition using Cohen’s d, along with the corresponding 95% confidence intervals, to quantify the magnitude and precision of the observed differences. We also conducted linear mixed models (LMMs) with participant ID as a random intercept to account for within-subject correlation. Covariates included age, sex, race, and randomization sequence. We implemented two complementary LMM strategies: 1. A 7-condition categorical LMM (primary analysis) and 2. A Factorial LMM excluding the mental task (secondary/mechanistic analysis).
All seven within-subject conditions were entered as a single categorical fixed factor. This approach provides a global assessment of condition effects and allows direct pairwise comparisons among any two conditions, while preserving participant-level clustering.
To test mechanistic hypotheses about immersion and auditory content, we fitted a second LMM restricted to the six auditory conditions with Immersion (immersive vs non-immersive), Auditory content (ocean, opera, pink noise), and their interaction as fixed effects. The mental task was excluded from this factorial model because it lacks auditory content; restricting the model in this way yields cleaner tests of immersion × sound interactions. Effect estimates are presented as estimated marginal mean differences with 95% confidence intervals. Pairwise contrasts and estimated marginal means (EMMs) were computed using the emmeans package and adjusted for multiple comparisons (Bonferroni). LMMs were estimated with maximum likelihood, which accommodates missing data under the missing-at-random assumption.

Missing repeated values were handled in the mixed-effects model. As a sensitivity analysis, we also ran multiple imputation (fully conditional specification with predictive mean matching, 40 imputations) for all outcomes and covariates.

Informed by prior findings that the first exposure to a condition may have the most salient effect on pain modulation ^21^, we chose to counterbalance the conditions by randomizing the delivery of the control VR and VR interventions. The sequences were created by running the MATLAB random function, assigning the *first* condition differently across participants. Moreover, the sequences were included as a covariate for all the analyses.

**Power calculation:** Power calculations were informed by effect-size estimates from our previous study using the same heat-pain paradigm and VR Ocean ^3^. In that study, immersion in VR Ocean increased pain-tolerance temperature by 1.025°C, and the pooled standard deviation across baseline and VR Ocean was approximately 2.53°C, corresponding to a standardized effect of Cohen’s d ≈ 0.41 (small–to–medium) ^3^. For the present study, we prespecified a conservative medium effect size of d = 0.50, consistent with Cohen’s recommendations and the magnitude observed previously.

Because the true within-subject correlation was unknown a priori, and because the study involved seven experimental conditions, covariate adjustment, and expected loss of physiological data in a subset of participants, we selected a conservative recruitment target of 61 participants. This target exceeds the most conservative estimate (ρ ≈ 0) and provides robust power and precision across the planned mixed-effects analyses.

Outliers. We controlled for outliers for each outcome using the Tukey formula as follows: *Upper*=Q3+(2.2*(Q3−Q1); *Lower*=Q1−(2.2*(Q3−Q1) whereby Q1 and Q3 were equal 25% and 75% percentiles respectively, to define upper and lower cut-offs. Results of regression and repeated measure ANCOVA analyses did not vary when the outliers were removed, or the full dataset was used.

SCR data analyses. Due to positive skewness in the SCR data, a square root transformation was applied to the AUC values to normalize their distribution. Repeated measures ANOVA were then conducted for each modality to examine the effects of condition on SCRs. When the assumption of sphericity was violated, Greenhouse-Geisser correction was applied to adjust the degrees of freedom. In terms of autonomic measurement (i.e., GSR), data from 25 participants was noisy and not analyzable. Therefore, the analysis for GSR was limited to 41 participants.

**Multi-level mediation models**

Given the observed net gain in heat pain tolerance, along with improvements in pain intensity, unpleasantness, mood, and anxiety, we conducted multilevel mediation analyses to examine whether the effects of VR on pain-related outcomes were mediated by changes in mood and anxiety. We used the Mlmed SPSS macro (Mlmed, Beta 2.^23,24^), which is appropriate for hierarchical data structures, where experimental conditions (Level 1) are nested within participants (Level 2).

For the 1-1-1 multilevel mediations, the two conditions (VR Ocean vs. Baseline) were entered as the independent variable (X), visual analog scale (VAS) ratings for mood, anxiety, and enjoyment, were entered as mediators, separately, and heat pain tolerance, VAS pain intensity, and VAS pain unpleasantness were each entered as the dependent variable (Y) in separate models. Participant ID was included as the cluster variable. Within-subject mediation effects were estimated using 95% bootstrapped confidence intervals (Cis). An indirect effect was considered significant if the 95% bootstrapped CI did not include zero.

All data analyses were conducted using SPSS version 24 (SPSS Inc., Chicago, IL, USA). The level of statistical significance was set at p = 0.05.

**Results**

**Demographic, psychological and clinical characteristics.** Among the 62 participants with TMD, most were women (66.1%) with a mean age of 34.7 years (range: 19–55). The majority identified as non-Hispanic or Latino (91.9%), and nearly half (48.4%) as White. Regarding socioeconomic status, 21.0% had a household income under $20,000, 48.4% were college graduates, and 69.4% were never married.

Clinically, participants reported moderate pain intensity (49.8 ± 2.8) and interference (31.3 ± 3.6), with 40.4% classified as having high-impact pain. Average pain duration was 125 months (range: 5–504). Psychologically, mean scores were 27.5 on the Beck Depression Inventory and 41.9 on the State-Trait Anxiety Inventory.

Participants scored relatively high on the Emotion Regulation Questionnaire. The mean score for cognitive reappraisal was 30.98 (SD = 0.78; range: 19–42), indicating a strong tendency to reinterpret emotional situations positively. In contrast, expressive suppression was lower, with a mean of 14.50 (SD = 0.71; range: 4–28), suggesting that participants were less likely to inhibit outward emotional expressions.

On the Barcelona Music Reward Questionnaire, participants reported high levels of emotional evocation (M = 16.37, SD = 0.31; range: 12–20), mood regulation (M = 17.00, SD = 0.26; range: 13–20), and social reward (M = 15.17, SD = 0.41; range: 6–20), reflecting a strong emotional and interpersonal connection to music. Scores for sensory-motor responses (M = 14.41, SD = 0.26; range: 9–20) and musical seeking (M = 12.06, SD = 0.35; range: 6–20) were also moderately high, indicating general engagement with the physical and exploratory aspects of music. Participants showed elevated levels of game addiction, with a mean score of 95.16 (scale maximum = 100), suggesting a high degree of engagement with gaming, which may shape how immersive or rewarding they perceive virtual environments like VR.

**Sequence effects:** Pairwise comparisons indicated that participants who received VR Ocean first showed significantly smaller improvement across conditions compared with those who received VR pink noise first (mean difference = −1.35, 95% CI [−2.50, −0.20], p = .022) and 2D pink noise first (mean difference = −1.35, 95% CI [−2.55, −0.15], p = .028), but not compared with 2D Ocean first (mean difference = −0.54, 95% CI [−1.78, 0.70], p = .384). Differences relative to VR opera first and WMT first were not statistically significant (all ps > .12). Results are shown in Suppl Figure 2.

**Suppl. Figure 2. Sequence effects.** Plots show the changes in pain tolerance limits based on the order of presentation of each condition.


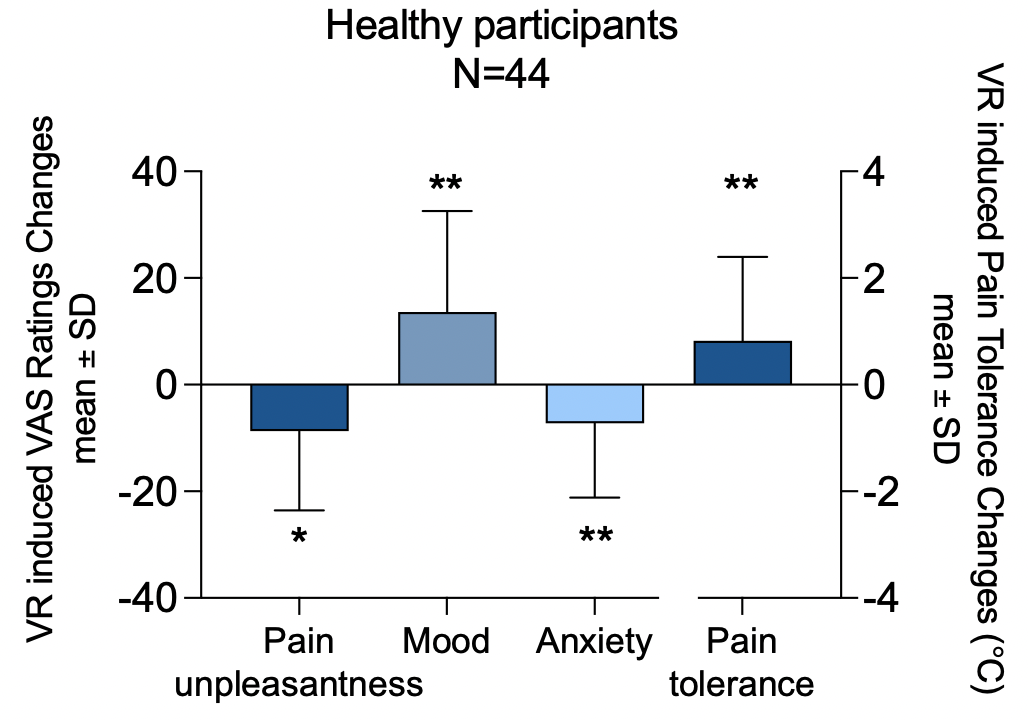

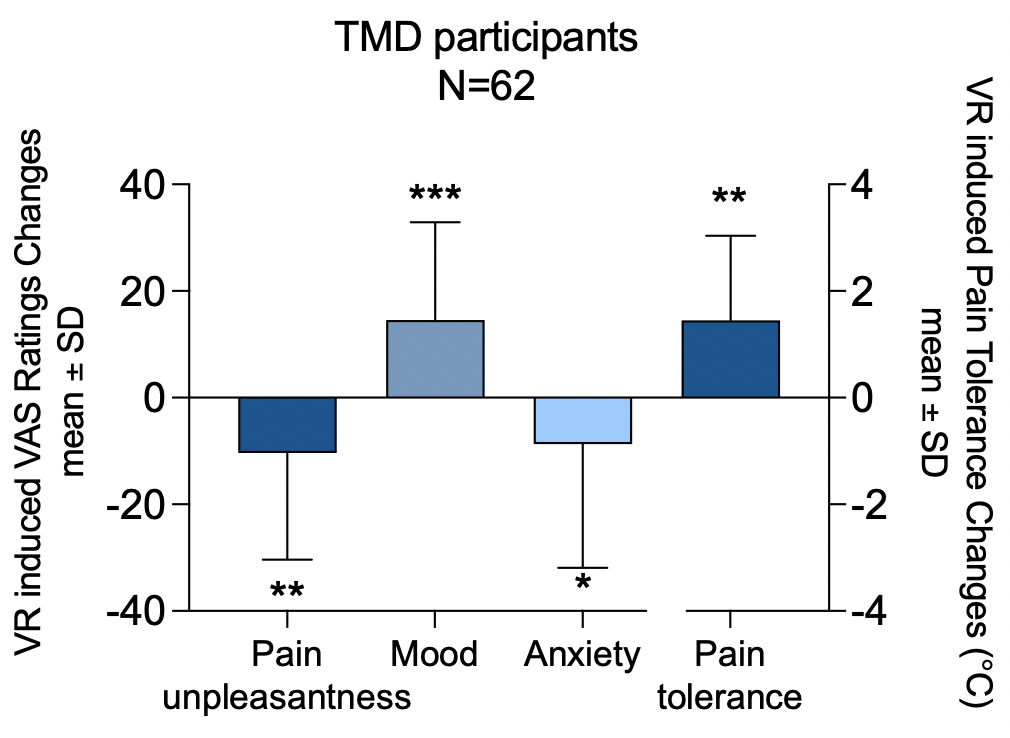


A

B

**Suppl. Figure 3. Comparison of VR effects between TMD participants with healthy controls.**

Across the experimental conditions, no significant group differences were found for pain unpleasantness, mood, or heat pain tolerance between healthy controls (A) and TMD (B). A significant group × condition interaction emerged for situational anxiety, with TMD participants showing elevated anxiety during the working memory task. No differences were observed in the VR-induced increase of pain tolerance.

**Suppl. Figure 4. Conceptual framework for VR-induced pain tolerance.**
This framework outlines that emotionally engaging audiovisual content such as ocean visuals and ambient music enhances VR-induced analgesia. Immersion alone does not drive analgesia; rather, affective enhancement mediates improvements in pain tolerance, mood, and anxiety. These findings support immersive, emotionally resonant VR interventions for chronic pain management. Bold words indicate the relevant factors.


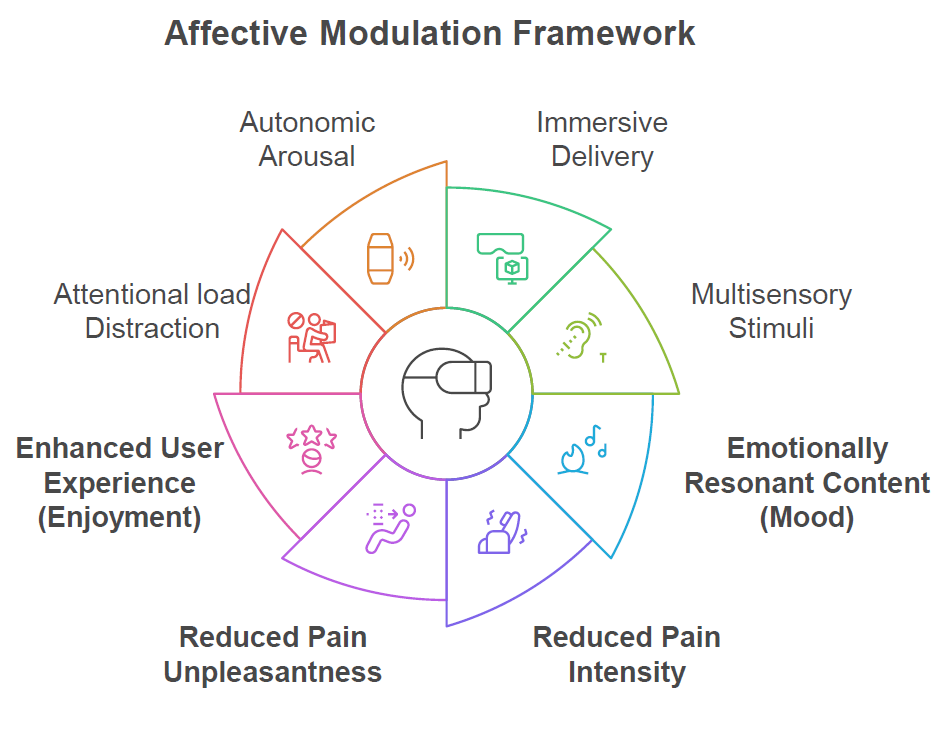


**References**

1. Schiffman E, Ohrbach R, Truelove E, et al. Diagnostic Criteria for Temporomandibular Disorders (DC/TMD) for Clinical and Research Applications: recommendations of the International RDC/TMD Consortium Network* and Orofacial Pain Special Interest Groupdagger. *J Oral Facial Pain Headache* 2014; **28**(1): 6-27.

2. Zakrzewska JM. Differential diagnosis of facial pain and guidelines for management. *Br J Anaesth* 2013; **111**(1): 95-104.

3. Dixon D, Pollard B, Johnston M. What does the chronic pain grade questionnaire measure? *Pain* 2007; **130**(3): 249-53.

4. Von Korff M, Dworkin SF, Le Resche L. Graded chronic pain status: an epidemiologic evaluation. *Pain* 1990; **40**(3): 279-91.

5. Dworkin SF, Huggins KH, Wilson L, et al. A randomized clinical trial using research diagnostic criteria for temporomandibular disorders-axis II to target clinic cases for a tailored self-care TMD treatment program. *J Orofac Pain* 2002; **16**(1): 48-63.

6. Maixner W, Fillingim RB, Williams DA, Smith SB, Slade GD. Overlapping Chronic Pain Conditions: Implications for Diagnosis and Classification. *J Pain* 2016; **17**(9 Suppl): T93-T107.

7. Ohrbach R, Larsson P, List T. The jaw functional limitation scale: development, reliability, and validity of 8-item and 20-item versions. *J Orofac Pain* 2008; **22**(3): 219-30.

8. Ohrbach R, Markiewicz MR, McCall WD, Jr. Waking-state oral parafunctional behaviors: specificity and validity as assessed by electromyography. *Eur J Oral Sci* 2008; **116**(5): 438-44.

9. Beck AT, Steer RA, Carbin MG. Psychometric properties of the Beck Depression Inventory: Twenty-five years of evaluation. *Clinical psychology review* 1988; **8**(1): 77-100 %@ 0272-7358.

10. Tran TD, Tran T, Fisher J. Validation of the depression anxiety stress scales (DASS) 21 as a screening instrument for depression and anxiety in a rural community-based cohort of northern Vietnamese women. *BMC Psychiatry* 2013; **13**: 24.

11. Barker HR, Jr., Wadsworth AP, Jr., Wilson W. Factor structure of the State-Trait Anxiety Inventory in a nonstressful situation. *J Clin Psychol* 1976; **32**(3): 595-8.

12. Wadsworth AP, Jr., Barker HR, Barker BM. Factor structure of the State-Trait Anxiety Inventory under conditions of variable stress. *J Clin Psychol* 1976; **32**(3): 576-9.

13. Keogh E, Reidy J. Exploring the factor structure of the Mood and Anxiety Symptom Questionnaire (MASQ). *Journal of personality assessment* 2000; **74**(1): 106-25 %@ 0022-3891.

14. Gross JJ, John OP. Individual differences in two emotion regulation processes: implications for affect, relationships, and well-being. *Journal of personality and social psychology* 2003; **85**(2): 348.

15. Mas-Herrero E, Marco-Pallares J, Lorenzo-Seva U, Zatorre RJ, Rodriguez-Fornells A. Individual differences in music reward experiences. *Music Perception: An Interdisciplinary Journal* 2012; **31**(2): 118-38.

16. Gentile D. Pathological video-game use among youth ages 8 to 18: a national study. *Psychol Sci* 2009; **20**(5): 594-602.

17. Benedek M, Kaernbach C. A continuous measure of phasic electrodermal activity. *Journal of neuroscience methods* 2010; **190**(1): 80-91.

18. Mischkowski D, Palacios-Barrios EE, Banker L, Dildine TC, Atlas LY. Pain or nociception? Subjective experience mediates the effects of acute noxious heat on autonomic responses. *Pain* 2018; **159**(4): 699-711.

19. Weaver KR, Griffioen MA, Klinedinst NJ, et al. Quantitative Sensory Testing Across Chronic Pain Conditions and Use in Special Populations. *Front Pain Res (Lausanne)* 2021; **2**: 779068.

20. Benedetti F, Mayberg HS, Wager TD, Stohler CS, Zubieta JK. Neurobiological mechanisms of the placebo effect. *J Neurosci* 2005; **25**(45): 10390-402.

21. Colloca L, Raghuraman N, Wang Y, et al. Virtual reality: physiological and behavioral mechanisms to increase individual pain tolerance limits. *Pain* 2020; **161**(9): 2010-21.

22. Faul F, Erdfelder E, Lang AG, Buchner A. G*Power 3: a flexible statistical power analysis program for the social, behavioral, and biomedical sciences. *Behavior research methods* 2007; **39**(2): 175-91.

23. Hayes AF, Rockwood NJ. Conditional process analysis: Concepts, computation, and advances in the modeling of the contingencies of mechanisms. *American Behavioral Scientist* 2020; **64**(1): 19-54.

24. Hu X, Zheng J, Fan T, Su N, Yang C, Luo L. Using multilevel mediation model to measure the contribution of beliefs to judgments of learning. *Frontiers in psychology* 2020; **11**: 637.
