# Supplementary material for: A critical role of affective content in the analgesic effect of virtual reality: a cross-sectional within-subject study
Source: Lancet Reg Health Am. 2026 Feb 16;55:101385. doi: 10.1016/j.lana.2026.101385 (PMC12930066; doi:10.1016/j.lana.2026.101385)
Supplement: Supplementary captions [file mmc2.docx]

**Supplementary Methods (Extended Methods)**

Full methodological details including recruitment strategy, diagnostic criteria and clinical assessments for temporomandibular disorder (TMD), additional questionnaires, experimental procedures, VR/2D condition descriptions and timeline, outcome definitions, sociodemographic measures; statistical analysis plan (RM-ANOVA and mixed-effects models, covariates, multiple comparison adjustments, effect sizes); handling of missing data and multiple imputation; randomization/sequence handling; power calculation; outlier handling; SCR preprocessing and analysis; and multilevel mediation models.

**Supplementary Figure 1. Participant flow diagram.**

CONSORT-style diagram showing screening (N=78), enrollment (n=68), completion (n=66), and analytic samples for (a) primary outcome (n=62), (b) secondary behavioral outcomes (n=62), and (c) physiological/SCR outcomes (n=41). Sex counts are provided at each stage.

**Supplementary Figure 2. VR effects in TMD vs healthy controls.**

Across conditions, no group differences for pain unpleasantness, mood, or heat pain tolerance; a group × condition interaction appears for situational anxiety with higher anxiety during the working-memory task in TMD. VR-induced increases in pain tolerance do not differ between groups.

**Supplementary Figure 3. Conceptual framework for VR-induced analgesia.**

Schematic illustrating that emotionally engaging audiovisual content (e.g., ocean visuals with ambient music) enhances VR analgesia. Immersion alone is insufficient; affective enhancement links VR exposure to increases in pain tolerance and improvements in mood/anxiety.

**Abbreviations:** VR, virtual reality; 2D, non-immersive/control display; TMD, temporomandibular disorder; SCR, skin conductance response; WMT, working-memory task.
